# Editorial Comment: Urologic surgery laparoscopic access: vascular complications

**DOI:** 10.1590/S1677-5538.IBJU.2015.0369.1

**Published:** 2017

**Authors:** Nikhil Sapre, Homayoun Zargar

**Affiliations:** 1 Royal Melbourne Hospital, Melbourne, VIC, Australia;; 2 Australian Prostate Cancer Research Centre, Melbourne, VIC, Australia

Branco et al. (1) describe two cases of injuries during laparoscopic renal surgery; both recognized immediately and repaired laparoscopically. Laparoscopic entry complications are an uncommon but potentially life-threatening complication of laparoscopic surgery. Most such injuries occur during initial trocar insertion and most commonly involve major vessels and/or bowel. Failing to recognize these immediately is a leading cause of death in patients (2). A review by the U.S. Food and Drug Administration (FDA) committee found that there was insufficient evidence to recommend any particular access technique over the others (veress needle, direct trocar and hasson) largely due to poor centralized reporting of these complications (3). Whilst laparoscopic renal surgery is safe even in patients with previous abdominal surgery (4), surgeons must be experienced with access techniques, select patient appropriately for laparoscopic procedures, be familiar with trocar designs, use safe trocar insertion techniques and be vigilant for injuries during access. Whilst some of these injures may be amenable to laparoscopic repair, one should have a low threshold for conversion to open and seeking appropriate help (general or vascular surgeon) when necessary to manage these promptly and safely.

Homayoun Zargar, MD Department of Urology Royal Melbourne Hospital 300 Grattan St, Parkville VIC 3050, Australia E-mail: Homi.zargar@gmail.com
